# Metabolic Considerations in Direct Procurement and Perfusion Protocols with DCD Heart Transplantation

**DOI:** 10.3390/ijms25084153

**Published:** 2024-04-09

**Authors:** Maria Arnold, Peter Do, Sean M. Davidson, Stephen R. Large, Anja Helmer, Georgia Beer, Matthias Siepe, Sarah L. Longnus

**Affiliations:** 1 Department of Cardiac Surgery, Inselspital, Bern University Hospital, University of Bern, 3010 Bern, Switzerland; 2 Department for BioMedical Research, University of Bern, 3008 Bern, Switzerland; 3 The Hatter Cardiovascular Institute, University College London, London WC1E 6HX, UK; 4 Royal Papworth Hospital, Biomedical Campus, Cambridge CB2 0AY, UK; 5 Graduate School for Cellular and Biomedical Sciences, University of Bern, 3012 Bern, Switzerland

**Keywords:** heart transplantation, donation after circulatory death, cardiac energy metabolism, warm ischemia, ischemia–reperfusion injury, ex situ heart perfusion, ex vivo heart perfusion, cardioprotection, reperfusion therapies

## Abstract

Heart transplantation with donation after circulatory death (DCD) provides excellent patient outcomes and increases donor heart availability. However, unlike conventional grafts obtained through donation after brain death, DCD cardiac grafts are not only exposed to warm, unprotected ischemia, but also to a potentially damaging pre-ischemic phase after withdrawal of life-sustaining therapy (WLST). In this review, we aim to bring together knowledge about changes in cardiac energy metabolism and its regulation that occur in DCD donors during WLST, circulatory arrest, and following the onset of warm ischemia. Acute metabolic, hemodynamic, and biochemical changes in the DCD donor expose hearts to high circulating catecholamines, hypoxia, and warm ischemia, all of which can negatively impact the heart. Further metabolic changes and cellular damage occur with reperfusion. The altered energy substrate availability prior to organ procurement likely plays an important role in graft quality and post-ischemic cardiac recovery. These aspects should, therefore, be considered in clinical protocols, as well as in pre-clinical DCD models. Notably, interventions prior to graft procurement are limited for ethical reasons in DCD donors; thus, it is important to understand these mechanisms to optimize conditions during initial reperfusion in concert with graft evaluation and re-evaluation for the purpose of tailoring and adjusting therapies and ensuring optimal graft quality for transplantation.

## 1. DCD as a Solution to Cardiac Graft Shortage

For patients with end-stage heart failure, heart transplantation is the most effective treatment to improve the quality of life and survival [1]; however, insufficient graft supply has become a critical obstacle. Conventionally, cardiac grafts for transplantation are obtained with donation after brain death (DBD). Unfortunately, the number of patients on waiting lists for heart transplantation far exceeds the number of suitable donor organs [2]. As a consequence, the shortage of cardiac grafts has become a major problem [3,4], and mortality for patients on the waiting list is high.

Widespread adoption of donation after circulatory death (DCD) in heart transplantation is expected to provide benefits in organ availability and will help to diminish waiting list mortality. Indeed, the feasibility of adult heart transplantation with DCD has been demonstrated with excellent results [5,6,7,8,9]. Notably, patient outcomes are similar to those with conventional donation after brain death at six-month [10] and one-, four-, and five-year time points [9,11,12]. Furthermore, with the addition of DCD, a 15–48% increase in adult heart transplant activity has been observed [9,11].

Despite these extremely promising results, clinical protocols for DCD heart transplantation remain to be optimized, as cardiac grafts from DCD donors are exposed to conditions different from those in conventional DBD. For example, a DBD heart is perfused until organ procurement, whereas, in DCD, the heart undergoes a period of warm, global, no-flow ischemia between circulatory arrest and procurement [3]. Although hearts undergo ischemia in both DBD and DCD, DBD hearts are first protected by cooling and cardioplegia, while with DCD, ischemia occurs under warm, unprotected conditions. Warm ischemia is of particular concern, as it can rapidly lead to severe and irreversible tissue damage. Furthermore, conditions surrounding circulatory arrest, such as increased circulating catecholamines, as well as increasingly severe cardiac hypoxia under working conditions are also likely to contribute to cardiac damage. As such, the procurement and preservation protocols established for DBD hearts are not optimal for DCD hearts.

Importantly, opportunities to intervene prior to procurement to improve the protection of organs for donation are limited for ethical reasons [13]. The Belgian protocol, for example, marks one exception, where antemortem interventions are not prohibited by law as long as they do not directly cause the patient harm or discomfort. This allows for antemortem heparin administration and insertion of a guidewire or cannula [14]. As such, clinically relevant techniques to optimize cardiac tolerance to warm ischemia, such as protective reperfusion strategies, must be identified; approaches that are best adapted for cardiac preservation during graft storage must be developed for previously ischemic hearts, and reliable means to assess the viability of DCD grafts need to be established [4].

The introduction of ex situ heart perfusion (ESHP), used widely in DCD heart transplantation, is an emerging area that offers new possibilities for the application of strategies directed at cardioprotection, as well as graft evaluation. Optimization of the DCD procurement protocol was initially implemented with the addition of erythropoietin (EPO) and glycerin trinitrate (GTN) to the preservation solution [15], and further optimization has included the use of methylprednisolone; increased perfusate albumin; and flow-targeted, rather than pressure-targeted, ex situ perfusion [16]. These more recent changes led to a strong decline in the requirement for post-operative extracorporeal membrane oxygenation (ECMO) support reported by the Australian team, indicating that heart function following transplantation was improved [16].

In this review, we aim to bring together knowledge about changes in cardiac energy metabolism and its regulation that occur in the DCD donor during withdrawal of life-sustaining therapy, during circulatory arrest, and following the onset of warm ischemia. A better understanding of these changes and their implications in cardiac injury should help to optimize therapeutic strategies aimed to minimize tissue damage when interventions are possible and/or at the time of reperfusion.

## 2. Sequence of Events in the DCD Donor

**Figure 1:**
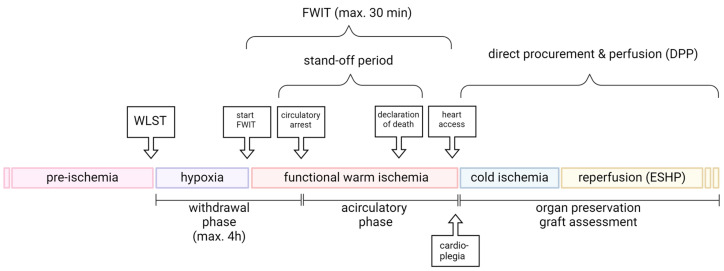
Sequence of events in direct procurement and perfusion clinical protocols for DCD heart transplantation with Maastricht category III donors. ESHP, ex situ heart perfusion; FWIT, functional warm ischemic time; WLST, withdrawal of life-sustaining therapy.

Several different conditions can be observed surrounding donation after circulatory death. Therefore, four main Maastricht categories for DCD donors have been defined [17]. The first category is dead on arrival, which includes, for example, victims of an accident outside the hospital. The second category is unsuccessful resuscitation, which includes patients brought into the hospital during resuscitation. The third category is awaiting cardiac arrest. It includes patients that are in the hospital and from whom the family has agreed to withdraw life-sustaining therapy (WLST). This category is also called controlled DCD because the circumstances and the time of warm ischemia are known and kept to a minimum. The fourth category is cardiac arrest while braindead. These patients suffer an unexpected cardiac arrest after brain death [17]. For DCD heart transplantations, generally, donors of the controlled Maastricht category III, in which the WLST is planned, are currently used [12,15,18]. Following WLST, circulatory arrest will ensue, and then a stand-off period must be awaited according to legal requirements and institutional policies. Only once the stand-off period has elapsed can the heart be accessed by the surgeon. Protocols differ with respect to heart procurement and assessment, with thoraco-abdominal-normothermic regional perfusion (ta-NRP) in the donor, or direct procurement and perfusion (DPP). The sequences of events in the donor up until accessing the heart are similar in DPP and ta-NRP; however, opportunities for managing conditions and therapeutic interventions at reperfusion are more limited in ta-NRP. For the purposes of this review, we will concentrate on the direct procurement and perfusion protocol (DPP; Figure 1). In this context, the main concern for DCD organs is damage incurred during the withdrawal (before circulatory arrest); during warm ischemia; and upon normothermic, oxygenated reperfusion with ESHP.

### 2.1. Withdrawal Phase

In a Maastricht category III donor, the organs are subjected to multiple systemic changes before and during the process of dying. Typically, these donors do not meet formal brain death criteria prior to WLST, but have non-recoverable neurologic injury [19]. The withdrawal phase consists of the time between the moment when life-sustaining therapy is withdrawn and the moment when circulatory/cardiac arrest is declared [20].

The duration of the withdrawal phase can vary substantially among donors, as teams await progression to circulatory arrest for differing periods according to local regulations. For example, this period ranges from approximately 1.5 h in Australia and Austria to 4 h in the UK; if circulatory arrest does not occur within these times, the transplant team will stand down [16]. For the 78% of potential donors who progress to circulatory arrest within the acceptable durations, the time from WLST to asystole is approximately 10 min in Australia [11] and 12 min in the UK [9].

### 2.2. Warm Ischemic Time (WIT)

Despite the lack of consensus on FWIT definitions, the sequences of events affecting cardiac energy metabolism during DCD organ donation remain the same. There is currently no standardized definition for warm ischemic time (WIT) in clinical practice. Given the potential for hemodynamic instability following WLST, some centers consider WIT to start at WLST [16]. Accordingly, the use of functional warm ischemic time (FWIT) has been introduced for a more precisely defined organ ischemia, and is considered to start when decreases in the donor fall below the predefined values for systolic blood pressure (usually 90–50 mmHg) or oxygen saturation (70%) [16]. Furthermore, the end of WIT is considered to coincide with the administration of cardioplegia by some, or with oxygenated blood reperfusion by others [11,12]. As the tolerance of the heart to warm ischemia is very limited, clinical data supports limiting FWIT to a maximum of 30 min [4,21]. The median FWIT duration was reported by the Papworth DCD center to be 24 min (range: 8–49) [9], and the mean FWIT duration was reported by the St. Vincent’s DCD center to be 22 min (range: 14–35) [16]. Thus, potential donor hearts procured using the DPP protocol are subjected to similar conditions, regardless of the precise FWIT definition.

### 2.3. Hemodynamic Changes

Much insight into hemodynamic changes in DCD donors can be gained from preclinical research. White et al. [22] categorized the hemodynamic changes occurring following WLST in a porcine model into different phases. The first 1.5 min following WLST was called the *pulmonary vasoconstriction phase*, during which oxygen partial pressure, cardiac output, and systemic oxygen delivery decrease, accompanied by a decline in left ventricular function and systemic vascular resistance. In the *hyperdynamic phase* (1.5–4 min), heart rate, biventricular function, cardiac output, and systemic vascular resistance increase, and systolic aortic pressure rises. During the *agonal phase* (4–7 min), a rapid decline in cardiac output and biventricular function occurs [22]. At 7–8 min, central venous pressure reaches levels similar to mean arterial pressure and is therefore termed the *circulatory arrest phase*. Also, in a porcine DCD model, Iyer et al. reported similar phases, with an immediate and progressive fall in systemic arterial pressure after WLST, a hyperdynamic phase after the first 2–3 min, and central venous pressure increasing in the first five minutes [23]. Similar patterns have been observed in DCD rat models, with a hemodynamic compensation after WLST followed by progressive hypotension and, ultimately, circulatory arrest [24,25]. However, the time from WLST to circulatory arrest is substantially shorter in rat models compared to pig models (3–5 min [24,25] vs. 7–8 min [22,23], respectively).

## 3. Metabolic Changes in the DCD Donor and Their Impacts on the Heart

Few reports have described the (patho)physiologic changes that occur in humans following WLST until circulatory arrest; however, preclinical models of DCD provide some information [22,23,24,25,26].

After the hyperdynamic period, hypoxia is followed by a decrease in arterial blood pressure and pulsatility, resulting in cardiac ischemia when cardiac perfusion is insufficient to prevent the metabolic shift from aerobic to anaerobic glycolysis [27]. Indeed, with the start of warm cardiac ischemia defined as systolic blood pressure < 50 mmHg, coronary hypoperfusion and myocardial ischemia are ostensibly already ongoing [23]. Dramatic changes in cardiac metabolism and function develop as a result of oxygen demand exceeding its supply [28]. The main ATP production machinery, oxidative phosphorylation, ceases, and the aerobic–anaerobic metabolic transition occurs [27,29,30].

### 3.1. Hypoxia

Partial oxygen pressure and systemic oxygen delivery decline rapidly following WLST and a hyperdynamic period ensues [22,23,24,25], which results in exposure of the heart to potentially damaging conditions in the progression to FWIT. In human DCD, the course and timing of progression to hypoxia following WLST is variable [16]; however, all hearts will be exposed to hypoxia, even if only briefly, prior to the start of FWIT. In preclinical DCD models, progression to hypoxia occurs within the first minute following WLST and continues for approximately 4–6 min until the start of FWIT (according to the definition of SAP < 50 mmHg), resulting in the heart being under hypoxic conditions for approximately 6 min before circulatory arrest [22,23].

Hypoxia leads to the accumulation of hypoxia-induced factor 1 (HIF1), which promotes glycolysis. The effects of acutely increased pre-ischemic glycolysis rates on subsequent graft quality have not been investigated in the context of DCD heart transplantation; however, they would be expected to decrease the myocardial glycogen content prior to global warm ischemia, which could be protective for the heart by limiting acidosis during ischemia and the resulting Na^+^ and Ca^2+^ overload [31]. Importantly, HIF-regulated pathways tune the balance of metabolic pathways to provide ATP and activate cell-survival pathways, and, therefore, help to protect the heart against hypoxia [32]. Hypoxia may, therefore, also exert protective effects, as it could stimulate pathways activated in pre-conditioning. However, reported protocols with hypoxic pre-conditioning generally require intermittent application by several (short) cycles of hypoxia and reperfusion prior to the index ischemia [33].

### 3.2. Catecholamines

Catecholamines are rapidly released into the circulation in response to hypoxia. Indeed, in DCD pig and rat models, steep increases in both circulating adrenaline and noradrenaline occur within the first 4 min post-WLST and continue to rise during FWIT [22,23,25]. In turn, sympathetic activity leads to rapid catecholamine (noradrenaline, adrenaline, and dopamine) increases in the myocardial interstitial fluid [34,35,36]. The effects of catecholamines on cardiac metabolism are numerous, including increased intracellular and mitochondrial calcium; mobilization of glycogen stores; as well as stimulation of glycolysis, beta-oxidation, and lipid accumulation, as reviewed by Wells and colleagues [37]. Importantly, adrenaline treatment before ischemia has been reported to reduce post-ischemic recovery of the rat myocardium, possibly as a result of exacerbated calcium overload [38]. Furthermore, catecholamine-induced increases in circulating energy substrates (glucose, free fatty acids) likely also contribute to altered cardiac energy metabolism prior to FWIT. Notably, the effects of increased circulating glucose have not been investigated; however, the acute effects of high circulating fatty acids lead to reduced post-ischemic recovery [39].

Sympathetic activity of the heart is closely related to the progression of cell injury in myocardial ischemia. Efferent sympathetic nerves are activated in early ischemia due to a fall in cardiac output and blood pressure. Adenosine, which is formed in the ischemic myocardium, suppresses the exocytosis of noradrenaline and prevents its excessive accumulation. However, with the progression of ischemia beyond 10 min, the myocardium is no longer protected and its release increases, leading to excessive interstitial noradrenaline concentrations. Furthermore, myocardial ischemia increases the number of β-adrenergic receptors, which causes enhanced catecholamine sensitivity in the heart during early ischemia. Within 20 min of ischemia, the extracellular noradrenaline concentration in the heart is 100–1000 times higher than normal, and the combination of enhanced responsiveness to catecholamines with extremely high concentrations of noradrenaline is believed to accelerate irreversible cell damage [40]. Importantly, very high catecholamine concentrations lead to sympathetic overstimulation, which contributes to systemic changes in energy metabolism, for example, hyperglycemia, insulin resistance, and elevated levels of circulating free fatty acids [41].

### 3.3. Carbohydrate Metabolism

After WLST, circulating lactate levels progressively increase and profound acidosis develops [22,23,25]. In addition, we have recently measured statistically significant increases in glycemia during the progression from WLST to FWIT in a porcine model of DCD [42].

Although increased pre-ischemic levels of circulating lactate reduce cardiac rates of glycolysis and fatty acid oxidation [43], the effects on post-ischemic cardiac recovery are not well studied. In an isolated rat heart model of DCD, brief exposure of hearts to increased pre-ischemic lactate (1 mM) significantly reduced cardiac recovery, as determined by ventricular function and release of cell death markers, compared to hearts exposed to physiologic pre-ischemic lactate (0.5 mM). Greater post-ischemic mitochondrial damage and calcium overload in hearts with higher pre-ischemic lactate levels provide a potential mechanism for the reduced cardiac recovery [44].

Lactate has long been considered simply as a by-product of glucose metabolism; however, it is now believed to play an important role as a signaling molecule (see detailed description below) [45]. Although lactate is a substrate for oxidative phosphorylation [45], during global ischemia, its role is limited. Thus, DCD conditions will result in high circulating lactate levels prior to ischemia, and further generation of lactate will occur during warm ischemia by the heart itself.

As a result of limited oxygen availability during ischemia, the rates of glucose and fatty acid oxidation (FAO) rapidly decrease. As ATP demand quickly exceeds production levels, the concentration of intracellular ADP and AMP rises. High AMP and low ATP levels promote 5′-AMP-activated protein kinase (AMPK) activity to increase GLUT translocation to the plasma membrane and stimulate glucose transport and glycolysis [46,47]. Additionally, glycogen stores are mobilized to support glycolysis [48]. Because glucose oxidation “uncouples” from glycolysis and FAO decreases, the heart becomes a net lactate producer and accumulates H^+^, resulting in intracellular acidosis [47,48]. Glycolysis–glucose oxidation uncoupling, as well as diminished energy production and nutrient availability, dysregulate ion concentrations, leading to H^+^, potassium, sodium, and calcium overload [47]. Importantly, with extended ischemia, glycolysis will be inhibited by its accumulated products (lactate, protons, and NADH) [49,50].

### 3.4. Fat Metabolism

In Maastricht category III DCD donors, acute, high levels of circulating free fatty acids are expected as a result of increased circulating adrenaline and heparin administration (when permitted) [39]. The first indication of circulating free fatty acids levels in a rat DCD model reported concentrations of 1.22 ± 0.69 mmol/L at FWIT [25], representing an approximately three-fold increase compared to reported physiologic levels [51]. Our group has also recently measured similar increases in a porcine DCD model, with an approximate four-fold increase between pre-surgical and FWIT levels [42].

Conditions or treatments affecting circulating free fatty acids may impair myocardial metabolism during hypoxia, ischemia, and reperfusion; [52] for example, high levels of free fatty acids will impair glycolysis and glucose oxidation [50], and can influence cardiac recovery during reperfusion. In several pre-clinical studies, differences in cardiac metabolism and recovery after ischemia have been investigated in hearts from fasted and fed rats. Fasting elevates serum fatty acids and myocardial fatty acid uptake, promoting a substrate shift towards greater fatty acid use [53]. This increase in fatty acid oxidation (FAO) elevates the glycogen content through the inhibition of the glycolytic pathway [31,53,54,55]. Fed rats, on the other hand, present higher glucose and insulin concentrations in the blood, and, therefore, shift cardiac metabolism towards greater glucose oxidation [56,57]. However, the findings are inconsistent; while some report an increased post-ischemic cardiac recovery in fasted compared to fed rats [31,53,54,55,56], others claim the exact opposite [57,58]. In an isolated rat heart model of DCD, a brief pre-ischemic exposure to high levels of free fatty acids (1.2 mM palmitate) lowered post-ischemic functional recovery by 50% compared to glucose as the sole pre-ischemic energy substrate [39].

Importantly, high pre-ischemic levels of free fatty acids may predispose hearts to lipotoxicity upon reperfusion. During ischemia, secondary to the lack of oxygen availability for mitochondrial oxidative phosphorylation and the resulting accumulation of reduced equivalents, FAO is decreased. Lipotoxicity can occur when the rates of fatty acid uptake exceed those of FAO, such as during ischemia, and results in the accumulation of toxic fatty acid metabolites such as acyl-CoA and acylcarnitine in the cytosol and mitochondrial matrix [34,35,59]. In cardiac mitochondria, accumulation of long-chain acylcarnitines is proposed to occur during ischemia as a result of increased transport into the mitochondria (via activation of carnitine palmitoyl transferase 1), and decreased activity of carnitine palmitoyl transferase 2 as a result of reduced oxidative metabolism [59]. Accumulation of long-chain acylcarnitines leads to inhibition of mitochondrial oxidative phosphorylation, mitochondrial membrane hyperpolarization, and excess ROS production [59]. These intermediates can promote the formation of amorphous intramitochondrial densities and disruption of mitochondrial cristae, which may ultimately disrupt mitochondrial function [34]. Furthermore, accumulation of lipid species in the heart has been associated with structural myocardial damage, impaired myocardial contractility, cardiac fibrosis, inhibition of the insulin signaling pathway, activation of NFκB and inflammatory cytokine signaling, and cell death [60].

### 3.5. Ketone Metabolism

Although, under physiologic conditions, the majority of cardiac ATP is derived from fatty acids (40–60%) and carbohydrates (20–40%), ketone bodies (β-hydroxybutyrate, acetoacetate and acetone) may be used as alternative energy sources contributing 10–15% of ATP [61,62]. The heart metabolizes ketone bodies to acetyl-CoA, which acts as a substrate for the TCA cycle, and several recent studies have suggested that ketone bodies affect cardiac metabolism in pathologic conditions [61,62]. Increased circulating levels of ketones could be expected in DCD donors as a result of both increased catecholamines and administration of heparin [63,64,65]. Although increased ketone body oxidation may be considered beneficial by helping to maintain oxidative metabolism, it may also support increased mitochondrial protein acetylation, which compromises cardiac energetics [61]. Furthermore, ketone body oxidation may also lead to decreased oxidative metabolism through the depletion of tricarboxylic acid cycle intermediates (i.e., decreased anaplerosis) [66]. Importantly, high concentrations of acetate have been demonstrated to selectively impair fatty acid metabolism in isolated rat hearts [67], which may contribute to further accumulation of toxic fatty-acid intermediates in DCD grafts. In contrast, a recent study in mice showed that after ligation of the left anterior descending artery, administration of β-hydroxybutyrate at reperfusion reduced infarct size (by 50%), preserved cardiac function, and increased autophagy [68]. Given these reports of both beneficial and detrimental effects, further investigation of circulating ketones and cardiac ketone metabolism in the context of DCD is required in order to precisely understand their role in cardiac graft quality.

### 3.6. Branched-Chain Amino Acid Metabolism

Branched-chain amino acids (BCAAs; isoleucine, leucine, and valine) are essential amino acids that play a role in metabolic homeostasis. The catabolic pathway of BCAAs ends with the generation of acetyl-CoA and succinyl-CoA and their oxidation in the TCA cycle or anaplerosis [69]. In a healthy heart, BCAAs contribute 1–2% to the cardiac ATP production [37].

During ischemia, circulating and cardiac BCAAs accumulate, which leads to decreased pyruvate dehydrogenase (through a downregulation of the hexosamine biosynthetic pathway, decreasing O-linked N-acetylglucosamine (O-GlcNAc) modification of PDH) and, therefore, an inhibition of glucose oxidation [69]. As mentioned above, glycolysis–glucose oxidation uncoupling during reperfusion exacerbates H^+^, sodium, and calcium overload [47].

### 3.7. Cellular Signaling

As mentioned above, in addition to acting as energy substrates for the heart, metabolites and their intermediates also play key roles in cellular signaling and the regulation of many cellular processes. Importantly, several key energy sensing/metabolism regulating signaling pathways are triggered under conditions of metabolic stress, as occur in DCD, and possess wide-ranging effects, as described below.

AMPK is a stress signaling enzyme that orchestrates energy production by modulating anabolic and catabolic metabolic pathways [70,71]. It is activated following hypoxia or ischemia and is rapidly normalized upon reperfusion. This cellular energy sensor, also known as the “metabolic master switch”, and its mediators regulate diverse targets, e.g., Akt substrate of 160 kDa (AS160), protein kinase C (PKC), endothelial nitric oxide synthase (eNOS), and p38 mitogen-activated protein kinase/transforming growth factor β-activated protein kinase 1-binding protein complex (TAB) 1 [72,73]. They also promote energy-producing processes (glucose uptake, glycolysis, and fatty acid metabolism) while inhibiting energy-consuming processes (protein, glycogen, and fatty acid synthesis) [70,71,74].

In addition, lactate and hypoxia-inducible factor 1 (HIF-1), the master regulator of oxygen homeostasis, are mutually regulated, i.e., increased lactate levels promote HIF-1 stabilization and expression of HIF-1-induced genes, while HIF-1 stimulates glucose uptake and glycolysis and, thereby, lactate production [75,76,77].

### 3.8. Cardiac Status at Procurement

**Figure 2:**
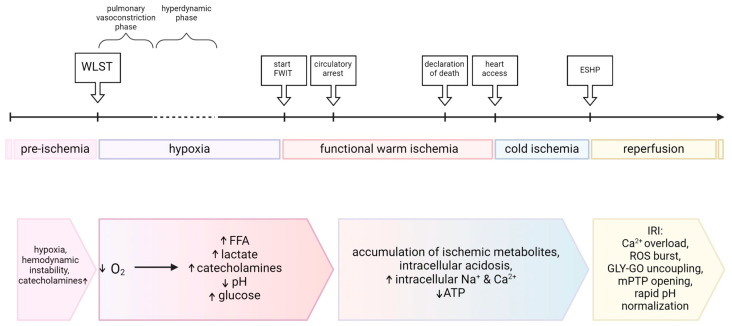
Metabolic changes during the sequence of events in direct procurement and perfusion clinical protocols for DCD heart transplantation. ATP: adenosine triphosphate; ESHP: ex situ heart perfusion; FFA: free fatty acids; FWIT: functional warm ischemic time; GLY: glycolysis; GO: glucose oxidation; IRI: ischemia–reperfusion injury; mPTP: mitochondrial permeability transition pore; ROS: reactive oxygen species; WLST: withdrawal of life-sustaining therapy.

There are differing levels of vulnerability and severity of metabolic changes induced by ischemia, depending on the region of the heart. Under aerobic conditions, levels of pyruvate, lactate, and ATP are uniform in the outer, middle, and inner regions of the heart [28,78]. However, after 30 s of ischemia, tissue lactate levels are elevated and tend to increase from the subepicardium to the subendocardium [28,78]. The first 15-min period after the onset of total ischemia is thought to be a guide value at which myocardial tissue can still be saved by restoring the blood flow with minimal cell death (Figure 2) [28,30]. When oxygenated blood flow is not restored within this reversible period of 15 min, irreversible injury concomitant with cell death begins [28,30]. One view of cell death during ischemia is the concept of a critical ATP level; once the concentration undershoots this threshold, vital cell functions cease [70]. Anaerobic glycolysis can provide some ATP at the beginning of ischemia [28,30], but by 40–60 min, anaerobic metabolism slows down markedly and ceases [30]. By this time, most ATP is also gone. The cessation of energy production and accumulation of metabolites along with histological changes represent crucial points in the irreversible damage of the myocardium [30].

As a consequence of energy demand not being met during ischemia, the adenine nucleotide pool is steadily depleted [27,79]. Decreased ATP production during ischemia compromises various cellular processes. For example, reduced activity of Na^+^/K^+^-ATPase, responsible for ionic homeostasis, results in intracellular Na^+^ overload [34]. In addition, Ca^2+^ released during contraction can no longer be taken up by sarcolemmal and sarcoplasmic reticulum Ca^2+^ ATPases; hence, intracellular Ca^2+^ accumulation occurs, while intracellular acidosis simultaneously blunts myofilament Ca^2+^ sensitivity [34]. As a result, ischemic cells cease their contractile function, but can remain viable for some time [30]. Concentrations of inosine and AMP peak at 20–30 min after ischemic onset [28,79], while hypoxanthine becomes the most abundant purine catabolite with longer periods of ischemia [27,79].

As mentioned in Section 2.2, a maximum period of 30 min of warm ischemia is considered acceptable for cardiac grafts in DCD transplantation, and has afforded excellent recipient outcomes. Furthermore, several successful cases of DCD heart transplantation have been reported with FWITs exceeding 30 min, suggesting that there may be room to extend our window of acceptable warm ischemic durations; however, a more precise mechanistic, pathophysiological understanding, as well as means to evaluate and treat cardiac grafts, are required. Indeed, severe metabolic dysregulation has occurred by the time of heart access and protective interventions are possible, which should be carefully managed to guarantee optimal graft quality. Importantly, DCD with DPP offers the opportunity to tightly control multiple aspects of the initial cardiac reperfusion, which is recognized to play a key role in the ultimate level of cardiac IR injury. However, the optimal conditions for the reperfusion of DCD hearts have yet to be defined.

## 4. Metabolic Considerations during Procurement and Early Reperfusion of Cardiac Grafts

### 4.1. Cold Ischemia

With DPP, functional warm ischemia of the cardiac graft is followed by a short period of cold static storage to prepare the heart for ESHP and prime the ESHP system. It is recognized that one major protective mechanism of cold preservation is reducing metabolic rates; however, it does not prevent ATP consumption completely [37]. To date, no consensus regarding the optimal composition of the initial cardioplegia in clinical DCD exists. In general, St. Thomas N°2 or Custodiol solutions supplemented with the cardioprotective agents erythropoietin (EPO) and glyceryl trinitrate (GTN) are used [15,80,81]. Both of these cardioplegic solutions were originally designed for DBD hearts and are therefore hyperkalemic in order to arrest the heart. Importantly, little information is available on the effects of these conditions on metabolic effects in cardiac grafts obtained with DCD. In these hearts, which are already asystolic and have undergone warm ischemia for approximately 30 min at the time of cardioplegia, oxidative metabolism is certainly already arrested, and glycolytic activity is likely limited. Thus, the cold ischemia may have a lower impact on the metabolism of DCD grafts, but high potassium levels may exacerbate calcium overload and therefore aggravate IRI and be detrimental for the vascular endothelium [19,82]. Preservation solutions should be reconsidered to minimize IRI and optimize the recovery of cardiac metabolism in the context of DCD heart transplantation.

### 4.2. Reperfusion Injury

Rapid oxygen reintroduction at the beginning of reperfusion leads to changes in mitochondrial metabolism, including membrane potential collapse, calcium overload, mitochondrial swelling, cytochrome c release, cellular membrane disruption, and, lastly, cell necrosis [41].

Upon reperfusion, FAO typically recovers rapidly, while glucose oxidation rates remain repressed. High rates of FAO inhibit glucose metabolism; importantly, FAO inhibits glucose oxidation to a greater extent than glycolysis, leading to further uncoupling between glycolysis and glucose oxidation [83]. Shifting energy substrate metabolism during early reperfusion away from FAO and towards glucose oxidation has been shown to improve post-ischemic contractile recovery and limit ischemia–reperfusion injury (IRI) [84,85,86]. Along these lines, differing cardioprotective approaches have been validated, for example, direct stimulation of glucose oxidation through the addition of dichloroacetate, an inhibitor of pyruvate dehydrogenase kinase (PDK) [83,87], or pyruvate itself, which also inhibits PDK and therefore stimulates pyruvate dehydrogenase and the coupling of glycolysis with glucose oxidation [88,89]. Furthermore, the inhibition of FAO with the addition of a malonyl-CoA decarboxylase (MCD) inhibitor [90], trimetazidine (an inhibitor of the β-oxidation enzyme ketoacyl-CoA-thiolase) [91], or etomoxir (an inhibitor of the carnitine palmitoyltransferase 1) [92] improves cardiac function after ischemia. Several beneficial effects are induced by promoting glucose metabolism. Firstly, glycolytically-derived ATP plays a critical role in ion homeostasis, whereas the ATP produced during glucose oxidation is mainly used for contraction [46,93]. Therefore, an optimal balance between glycolysis and glucose oxidation is essential for post-ischemic cardiac recovery, guaranteeing sufficient glycolysis to restore the ion homeostasis after ischemia, but remaining at a level that allows coupling between glycolysis and glucose oxidation to limit further H^+^ production [85]. Secondly, cardiac efficiency (cardiac work performed per amount energy consumed) is affected by alterations in energy metabolism [94]. Cardiac work requires ATP, and the type of energy substrate being utilized for ATP production differs in oxygen efficiency (ATP produced by oxygen consumed), with glucose being more efficient than fatty acids. In fact, one glucose molecule requires 6 O_2_ and produces 31 ATP by going through glycolysis and glucose oxidation (5.2 ATP/O_2_ consumed), whereas oxidation of one palmitate molecule requires 23 O_2_ and produces 105 ATP per molecule (4.6 ATP/O_2_ consumed) [94]. Accordingly, energy substrate preference is particularly important during early reperfusion, when oxygen may still be limited.

### 4.3. Challenges and Opportunities

Although DCD cardiac grafts have a significant impact on increasing donor heart availability, there are several hurdles that need to be overcome to adopt this technique globally and effectively, as reviewed by Large and colleagues [13]. The question of pretreatment in the donor is one of several points debated amongst ethicists, as is the possibility of restoring circulation in the donor for heart evaluation prior to procurement [13]. Regardless of the precise clinical protocol used, the tolerance of a human donor heart to the conditions of DCD donation and the resulting status of the graft should be thoroughly understood. Importantly, the use of DPP and ESHP offers a multitude of possibilities to optimize and tailor therapies. Indeed, the ideal perfusion conditions and perfusate composition remain to be established—particularly during the first minutes of oxygenated reperfusion, at which time there is great potential to limit or prevent IRI [95,96]. In parallel, it is crucial to develop reliable means for graft evaluation to enable informed therapeutic action, determine suitability for transplantation, and optimize graft quality.

In the setting of DCD heart transplantation, little attention has been given to differences in cardiac graft quality and ischemia–reperfusion tolerance between male and female donors. Importantly, several preclinical studies have demonstrated that pre-menopausal female rat hearts are more tolerant than male hearts in both cellular [97] and animal models [98] of IR and/or myocardial infarction. Furthermore, sex differences in energy metabolism and metabolic flexibility have been identified. Sexual dimorphism in myocardial acylcarnitine and triglyceride metabolism has been reported in non-obese diabetic (NOD) rats; male NOD rats exhibited an accumulation of triglycerides, and female NOD rats presented a lower triglyceride content together with an accumulation of acylcarnitines [99]. These sex differences in the expression of genes involved in lipid metabolism may contribute to the sexual dimorphism observed in diabetic cardiovascular disease [99]. Furthermore, these results indicate that lipid metabolism may be handled differently in the context of DCD, with acute high levels of free fatty acid exposure in males and females, and therefore influence graft quality. Several other reported cardiac sex differences are also likely to play a role in the context of DCD: post-ischemic glycolysis/glucose oxidation coupling [100,101], mitochondria size, coupling between oxygen consumption and ATP production, ROS production both under physiologic conditions and after ischemia–reperfusion [98,102], and mitochondrial calcium storage capacity [103]. However, one preclinical study reported no difference in infarct size between males and females in a rat model of DCD [104]. Nonetheless, sex-specific differences in DCD cardiac graft quality merit further investigation, and corresponding, tailored reperfusion treatments should be considered.

### 4.4. Potential Metabolic-Based Cardioprotective (Reperfusion) Strategies

Strategies to minimize cardiac ischemia and reperfusion injury have been extensively studied and are highly effective in animal models of myocardial infarction, although clinical translation to patients has been disappointing [105]. Despite this, potentially beneficial strategies should be reconsidered in DCD. Indeed, many of the identified obstacles for successful translation in acute myocardial infarction are absent or curtailed in DCD heart transplantation; for example, timely application of reperfusion strategies is a prerequisite, and co-medications and comorbidities are limited through donor selection. Furthermore, with DPP, perfusate composition and conditions can be tightly controlled and tailored to minimize IRI at reperfusion [19]. Additionally, during the time when the heart is undergoing ESHP, it can be aggressively treated without the potential detrimental impact of drugs or drug doses on other organ systems [19]. For example, the reported hepatotoxicity of etomoxir [106] can be disregarded in that context.

**Figure 3:**
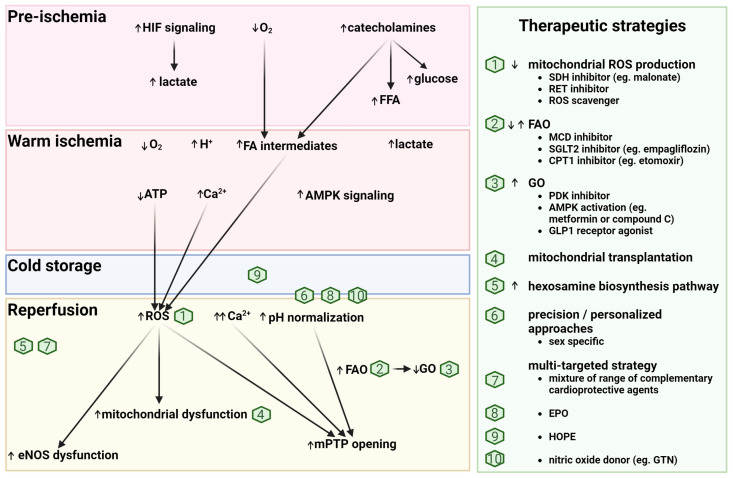
Potential metabolic-based cardioprotective reperfusion strategies. AMPK: 5′-AMP-activated protein kinase; ATP: adenosine triphosphate; CPT1: carnitine palmitoyl transferase 1; eNOS: endothelial nitric-oxide synthase; EPO: erythropoietin; FA: fatty acid; FAO: fatty acid oxidation; FFA: free fatty acids; GO: glucose oxidation; GLP1: glucagon-like peptide 1; GTN: glyceryl trinitrate; HIF: hypoxia-induced factor; HOPE: hypothermic oxygenated perfusion; MCD: malonyl-CoA decarboxylase; mPTP: mitochondrial permeability transition pore; PDK: pyruvate dehydrogenase kinase; RET: reverse electron transport; ROS: reactive oxygen species; SDH: succinate dehydrogenase; SGLT2: sodium glucose transporter 2.

Given the profound metabolic derangements, with critical implications for IRI, to which hearts are subjected during DCD [107], targeting metabolic pathways to optimize graft quality is a promising therapeutic approach (Figure 3). Indeed, it has been demonstrated that ESHP can help to normalize DCD-induced metabolic derangements in a porcine model [108]. One therapeutic targeting example is hypothermic, oxygenated perfusion (HOPE), which has been demonstrated to substantially improve cardiac recovery in preclinical models [109]. Importantly, oxygenated normothermic reperfusion immediately following ischemia, as occurs in DPP, is likely not optimal. In a DCD rat model, HOPE improved cardiac recovery after warm ischemia, and was most likely mediated through multiple mechanisms, including the preservation of mitochondrial function (via succinate oxidation) [109] and nitric oxide synthase activity [110]. Furthermore, HOPE may also help to deplete toxic fatty acid intermediates accumulated during ischemia before ESHP to reduce lipotoxicity. In addition, a substrate shift away from fatty acid metabolism and towards glucose metabolism in early reperfusion would lead to more efficient ATP generation [48]. Additional reports supporting metabolic targets to reduce acute IRI include the inhibition of the malate–aspartate shuttle, mitochondrial oxygen consumption, fatty acid oxidation, and mitochondrial succinate metabolism [50]. Succinate accumulates during ischemia, leading to mitochondrial ROS production during reperfusion via reverse electron transport through mitochondrial complex I [111]. Inhibition of this process with malonate is cardioprotective and would also be expected to improve cardiac recovery following DCD [112]. Indeed, in a rat model of DCD, the transient inhibition of mitochondrial complex I during early reperfusion led to reduced cardiac injury, likely due to decreased ROS production [113]. Also, an inhibitor of reverse electron transport (RET), which is a key mechanism for mitochondrial ROS production during reperfusion, could be used [111], or indeed, ROS scavengers. Furthermore, other studies in animal models have demonstrated that mitochondrial transplantation during reperfusion exerts cardioprotective effects by replacing damaged mitochondria with new, viable mitochondria. As a result, cellular respiration, oxygen uptake, and tissue ATP content are increased [114,115]. Additional reports highlight the potential benefits of metabolic targeting for cardioprotection, advocating for the utilization of SGLT2 inhibitors (e.g., empagliflozin). These inhibitors are noted for their ability to enhance palmitate uptake while reducing the accumulation of metabolites from incomplete fatty acid oxidation, thus contributing to a cardioprotective effect [116]. Transient activation of AMPK at reperfusion, with metformin or AICAR (5-amino-4-imidazolecarboxamide-riboside), may also be cardioprotective by increasing anaerobic glycolysis and, thus, providing energy to the heart. However, prolonged activation of AMPK will lead to increased acidosis and a decrease in cardiac recovery [117]. An increase in the hexosamine biosynthesis pathway leading to modification of proteins with O-linked attachment of N-acetylglucosamine moiety (O-GlcNAc) is cardioprotective, and can be achieved pharmacologically at the time of reperfusion [118]. Glucagon-like peptide-1 receptor agonists have also been shown to be cardioprotective, potentially through multiple effects, such as increasing cardiomyocyte glucose uptake, enhancing ATP synthesis, or modulating the activity of sodium–potassium adenosine triphosphatase [119].

Ultimately, a multi-targeted strategy may provide optimal cardioprotection [95]. For example, a cocktail of protective drugs (which includes ketone bodies and a range of other cytoprotective agents) has been used to preserve and recover cells in pig organs following 1 h of whole-body warm ischemia [120].

## 5. Limitations

There are limited data from our clinical experience as well as from pre-clinical research regarding the effects of DCD heart transplantation and the associated metabolic dysregulation on cardiac graft quality. As such, we have reported potential metabolic-based strategies that have mainly been tested in animal models of myocardial infarction. Further investigation is required in order to characterize metabolic changes and metabolic-based therapeutic strategies in the setting of DCD, both in large animal models and in clinical practice. Furthermore, research is lacking related to sex-specific differences and personalized approaches, which could be facilitated by the use of ESHP.

## 6. Conclusions

**Figure 4:**
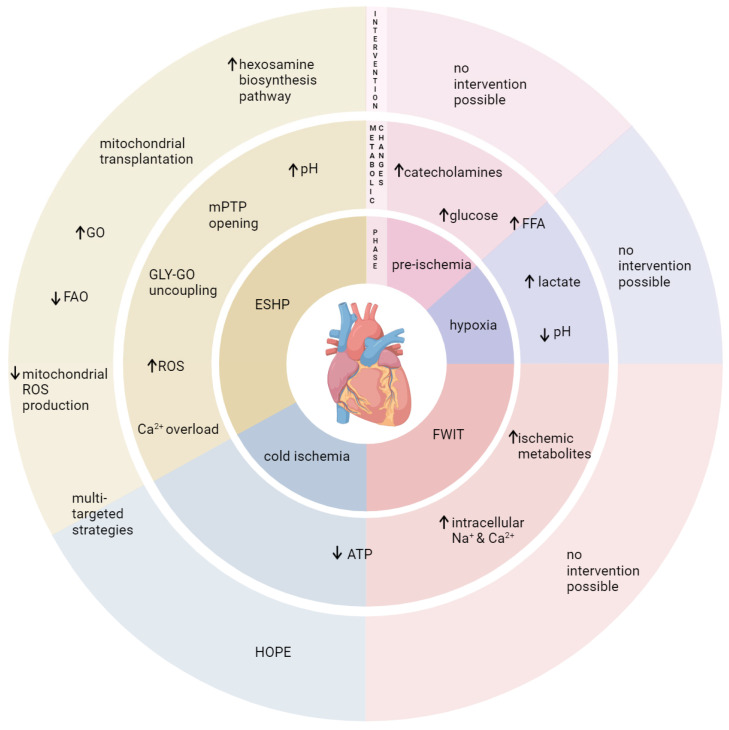
Metabolic changes and potential therapeutic approaches during the different phases of DCD heart transplantation. ATP: adenosine triphosphate; ESHP: ex situ heart perfusion; FAO: fatty acid oxidation; FFA: free fatty acids; FWIT: functional warm ischemic time; GLY: glycolysis; GO: glucose oxidation; HOPE: hypothermic oxygenated perfusion; mPTP: mitochondrial permeability transition pore; ROS: reactive oxygen species.

To conclude, acute metabolic, hemodynamic, and biochemical changes in DCD donors expose hearts to high circulating catecholamines, hypoxia, and warm ischemia, which could all negatively impact the heart and profoundly alter cardiac energy metabolism. Further metabolic changes and cellular damage occur with reperfusion. The altered energy substrate availability prior to organ procurement likely plays an important role in graft quality and post-ischemic cardiac recovery. Therefore, these aspects should be considered in clinical protocols as well as in pre-clinical DCD models (Figure 4). Notably, interventions prior to graft procurement are limited for ethical reasons in DCD donors; therefore, it is important to understand these mechanisms to optimize conditions during initial reperfusion in concert with graft evaluation and re-evaluation for the purpose of tailoring and adjusting therapies during ESHP, thereby ensuring the highest graft quality for transplantation. Concretely, we suggest (1) to reconsider the composition of the initial preservation solution (e.g., DCD-adapted solutions for pre-arrested, ischemic hearts); (2) to include a short period before ESHP with a hypothermic, oxygenated perfusion (HOPE); and (3) to optimize the reperfusion solution during ESHP (multi-targeted approach including shifting energy substrate metabolism towards glucose metabolism). Furthermore, personalized and sex-specific approaches should be considered in order to achieve both optimal graft quality and recipient outcomes.

## Data Availability

Not applicable.
